# Role of Erk5 expressed in bone marrow mesenchymal stem cells on bone homeostasis and its potential applications in cancer treatment

**DOI:** 10.18632/oncoscience.601

**Published:** 2024-05-20

**Authors:** Tetsuhiro Horie, Eiichi Hinoi

**Affiliations:** ^1^Medical Research Institute, Kanazawa Medical University, Kahoku, Ishikawa, Japan; ^2^Department of Pharmacy, Kanazawa Medical University Hospital, Kahoku, Ishikawa, Japan; ^3^Department of Bioactive Molecules, Pharmacology, Gifu Pharmaceutical University, Gifu, Japan; ^4^United Graduate School of Drug Discovery and Medical Information Sciences, Gifu University, Gifu, Japan; ^5^Center for One Medicine Innovative Translational Research, Division of Innovative Modality Development, Gifu University, Gifu, Japan

**Keywords:** bone marrow mesenchymal stem cell, extracellular signal-regulated kinase 5, Smad-specific E3 ubiquitin protein ligase 2, cancer, stem cell niche

Extracellular signal-regulated kinase 5 (Erk5) belongs to the mitogen-activated protein kinase (MAPK) family and is specifically phosphorylated and subsequently activated by MAPK/Erk5 kinase 5 (Mek5) [1]. Compared to classical MAPKs Erk1/2, Erk5 is unique in that it has distinctive structures on its C-terminus with two proline-rich domains and a nuclear localization signal domain, which regulate autophosphorylation and transcription. Erk5 signaling is activated by hypoxia, oxidative stress, cytokines and growth factors, and is involved in angiogenesis, neurogenesis and energy metabolism, as well as tumor growth and metastasis [2, 3].

Bone homeostasis is maintained by the coordinated activities of osteoclasts (bone-resorbing cells) and osteoblasts (bone-forming cells) derived from hematopoietic stem cells (HSCs) and bone marrow mesenchymal stem cells (BM-MSCs), respectively. Bone diseases such as osteoporosis and osteopetrosis are caused by an imbalance between bone resorption and formation due to aging, menopause, genetic mutations, etc. Previously, using paired-related homeobox 1 (*Prx1*)*-Cre*; *Erk5^fl/fl^* mice (MSC-specific *Erk5* knockout mice), we showed that Erk5 activates Smad-specific E3 ubiquitin protein ligase 2 (Smurf2) by directly phosphorylating Thr^249^ (Smurf2^T249^) and regulates skeletal development during embryogenesis by inducing the degradation of Smad1 protein, but the function of Erk5 in BM-MSCs in adulthood was not clear [4]. Therefore, we generated and analyzed conditional *Erk5* knockout mice (*LepR-Cre*; *Erk5^fl/fl^* mice) using leptin receptor (LepR)-Cre, which targets adult BM-MSCs [5, 6].

Micro-computed tomography (μCT) analysis revealed no significant changes in the femur of young (6-week-old) *LepR-Cre*; *Erk5^fl/fl^* mice, whereas marked BM ossification was observed at 12 weeks of age, and bone volume increased with growth. Histological analysis showed that femurs from *LepR-Cre*; *Erk5^fl/fl^* mice had increased osteoblasts and bone formation rate, while osteoclast activity was unchanged, indicating that abnormal ossification in adult *LepR-Cre*; *Erk5^fl/fl^* mice might be caused by enhanced osteogenesis rather than due to suppressed bone resorption.

Bioinformatic analyses indicated that BM-MSCs with low Erk5 expression/activity had higher osteoblast differentiation and osteogenic capacities. Subsequent *in vitro* experiments showed no difference in colony forming ability or expression of stem cell markers, while the expression of Runx2 and Osterix, master regulators of osteoblastogenesis, was markedly elevated in *Erk5*-deficient BM-MSCs. We examined whether Erk5/Smurf2 signaling is involved in these results and found that, as expected, phosphorylation levels of Smurf2^T249^ were significantly decreased in BM-MSCs derived from *LepR-Cre*; *Erk5^fl/fl^* mice. Furthermore, although no differences were observed in mRNA levels of Smad family (*Smad1*, *Smad4*, *Smad5*, *Smad6*, *Smad7* and *Smad8*), the protein levels of Smad1 and phospho-Smad1/5/8 were notably higher in *LepR-Cre*; *Erk5^fl/fl^* mice-derived BM-MSCs. This suggested that the loss of *Erk5* prevented Smurf2 activation and following degradation of Smad proteins by the ubiquitin-proteasome system.

Previously, we showed that the Smurf2^T249E^ mutant, in which the Thr^249^ residue was replaced with glutamate, mimicked the phosphorylation of Smurf2^T249^ and had activated E3 ubiquitin ligase activity [4]. In order to examine the Smurf2^T249E^ function *in vivo*, we generated Smurf2^T249E^ knock-in mice (*Smurf2^T249E/T249E^* mice) using CRISPR/Cas9 system, and no notable changes were observed in the femurs of these mice. We then evaluated the femurs of *LepR-Cre*; *Erk5^fl/fl^; Smurf2^T249E/T249E^* mice harboring *Smurf2^T249E/T249E^* knock-in allele by μCT and histological analyses and found that abnormal ossification in the BM of *LepR-Cre*; *Erk5^fl/fl^* mice was almost completely rescued. Given these results, we suggested that Erk5 in adult BM-MSCs is important for proper osteogenesis via phosphorylation of Smurf2^T249^.

Bone tissue not only plays a fundamental role in locomotion, protection of organ and storage for minerals (calcium and phosphate), but also has an endocrine function in secreting hormones, and osteocalcin (secreted by osteoblasts) serves a variety of roles, including regulation of energy metabolism [7]. In recent years, the association between osteocalcin and cancer has been studied, and it has been suggested that osteocalcin is relevant to bone metastasis of breast and prostate cancers [8]. Serum osteocalcin levels were significantly elevated in *LepR-Cre*; *Erk5^fl/fl^* mice, suggesting that Erk5 in BM-MSCs might be a new drug target to inhibit bone metastasis in these cancers [6].

Blood cells are produced by the differentiation of HSCs in the BM. LepR^+^ BM-MSCs, also referred to as Cxcl12-abundant reticular (CAR) cells, are major component of the microenvironment called HSC niche in adult BM and regulate HSC function by secreting several cytokines/chemokines such as Cxcl12 and Scf [9]. Proper bone formation is also associated with homeostasis of the hematopoietic system; for example, Seike et al. reported that CAR cell-specific *Ebf3* knockout mice showed remarkable increase in bone mass in the BM and abnormal hematopoiesis, which were caused by dysfunctions of CAR cells [10]. Although no significant abnormalities were observed in the immunophenotype of *LepR-Cre*; *Erk5^fl/fl^* mice under physiological states, it is possible that these mice show some phenotype in pathological conditions (e.g. acute myeloid leukemia) [6]. In addition, Erk5 expressed in chronic myeloid leukemia stem cells contributes to the maintenance of stemness and, intriguingly, it has been reported that bone mass decreases with leukemia [11, 12]. Erk5 inhibitors may therefore be an innovative therapeutic agent with dual benefits of suppressing leukemia stem cell function and improving the quality of life of leukemia patients by increasing bone mass, possibly leading to an improvement of the leukemia stem cell niche. We have also unveiled that MEK5/ERK5 axis is important for the self-renewal and tumorigenic potential of human glioma stem cells and that treatment with XMD8-92, a small molecule ERK5 inhibitor, suppresses glioblastoma progression [13]. Elucidating the function of Erk5 in cancer stem cells, cancer cells and their supporting microenvironment will contribute to a better understanding of cancer pathogenesis and the development of novel therapeutic strategies.
